# A Comparison of Dense and Sparse Optical Flow Techniques for Low-Resolution Aerial Thermal Imagery

**DOI:** 10.3390/jimaging8040116

**Published:** 2022-04-16

**Authors:** Tran Xuan Bach Nguyen, Kent Rosser, Javaan Chahl

**Affiliations:** 1 School of Engineering, University of South Australia, Mawson Lakes 5095, Australia; javaan.chahl@unisa.edu.au; 2 Aerospace Division, Defence Science and Technology Group, Edinburgh 5111, Australia; kent.rosser@dst.defence.gov.au; 3 Joint and Operations Analysis Division, Defence Science and Technology Group, Melbourne 3000, Australia

**Keywords:** optical flow, thermal imaging, LWIR, navigation, Unmanned Aerial Vehicles (UAVs), the image interpolation algorithm

## Abstract

It is necessary to establish the relative performance of established optical flow approaches in airborne scenarios with thermal cameras. This study investigated the performance of a dense optical flow algorithm on 14 bit radiometric images of the ground. While sparse techniques that rely on feature matching techniques perform very well with airborne thermal data in high-contrast thermal conditions, these techniques suffer in low-contrast scenes, where there are fewer detectable and distinct features in the image. On the other hand, some dense optical flow algorithms are highly amenable to parallel processing approaches compared to those that rely on tracking and feature detection. A Long-Wave Infrared (LWIR) micro-sensor and a PX4Flow optical sensor were mounted looking downwards on a drone. We compared the optical flow signals of a representative dense optical flow technique, the Image Interpolation Algorithm ($I^{2}A$), to the Lucas–Kanade (LK) algorithm in OpenCV and the visible light optical flow results from the PX4Flow in both X and Y displacements. The $I^{2}A$ to LK was found to be generally comparable in performance and better in cold-soaked environments while suffering from the aperture problem in some scenes.

## 1. Introduction

Robust navigation is a desirable capability for Unmanned Aerial Vehicles (UAVs). Many UAVs rely on the Global Position System (GPS) to sense their position. However, GPS requires a clear view of the sky to operate reliably, which limits its use in certain working environments such as in high-density urban areas, inside buildings, underground, or in areas where the signal is subject to deliberate or inadvertent interference. Additionally, the vertical error of GPS can be several meters on Earth due to the layers of the atmosphere, which refract and delay the transmission signals between the receivers and the satellites [1]. This issue makes GPS navigation-based techniques unreliable in confined spaces and indoors.

Unlike GPS-based navigation systems, vision-based systems do not rely on having an unobstructed view between the UAVs and the satellite. Instead, it relies on the on-board sensor, which can be an optical colour visual light sensor or a thermal sensor. Vision-based systems can provide real-time information about the surrounding dynamic environment and are resistant to conventional jamming. Hence, vision-based systems can be a good solution to aid UAVs to navigate in GPS-denied areas.

## 2. Related Work

There have been efforts to develop both active and passive vision-based techniques for navigation, yet a limited number of techniques have been attempted in darkness despite the potential to double the operating period. Thermal sensors are one imaging technology that can operate in darkness in some environments. There are many reasons for this, such as high cost and difficulty to acquire thermal sensors, limited access to airspace, and operational difficulties experienced at night [2].

Early researchers tried to utilise both visual and infrared spectrum to help robots navigating in dark and low visual environments.

Brunner et al. [3] combined the visual spectrum with infrared spectrum to aid the Simultaneous Localization And Mapping (SLAM) in low light and smoke situations. The result showed a significant increase in the SLAM performance in such conditions for unmanned ground vehicles. Papachristos et al. [4] developed a fusion of thermal and inertial sensor systems for small UAVs through a dark and GPS- denied environment. The study relied on a Long-Wave Infrared (LWIR) sensor to detect thermal objects, combined with the Support Vector Machine technique to accomplish real-time localisation. Another approach proposed by Khattak et al. [5] outlined a multi-modal fusion system from a fusion of visual light and the infrared spectrum combined with inertial sensors to help a small UAV to manoeuvre in a dark tunnel.

However, the previous approaches only utilised a rescaled 8-bit resolution instead of on full radiometric 14 bit. The reason for this approach is that the vast majority of open-source computer vision libraries such as OpenCV are designed with 8-bit processing in mind [6,7].

A rescaled 8-bit thermal frame results in lower contrast due to the loss of information due to 6 bits being discarded [8]. Furthermore, the study in [9] shows that with the same algorithm, utilising full input radiometric thermal information may produce better performance, and with less accumulated errors over time than its rescaled counterpart.

Khattak et al. [10] proposed a framework to use full radiometric 14-bit data from an LWIR sensor to navigate in a degraded visual environment. The framework was tested in an underground mine, demonstrating better results compared to a rescaled version. Recently, the same team [6] developed a thermal–inertial system for tracking features to determine a path for a small UAV with full 14-bit radiometric resolution.

These works have shown that thermal sensors can provide valuable information to navigate in low-light situations. However, these mentioned works are computationally demanding, which limits their use on small UAVs and in real-time applications. In contrast, biologically inspired optical flow techniques have been used by birds and insects to support them to navigate in cluttered environments [11]. Recent studies have shown that honeybees rely on optical flow for most of their navigation tasks, such as collision avoidance [12] or landing [13,14]. Optical flow is evidently an efficient and effective way to achieve more autonomous robust navigation for small UAVs.

Optical flow is defined as the apparent motion of image intensities or brightness patterns across multiple scenes [15]. The PX4Flow sensor [16] is one of the most widely used optical flow sensors, which has been integrated into many studies [17,18] with success. Optical flow can be used for active navigation, such as frontal object avoidance [19], to calculate time to impact [20] or can be used passively to collect information about the current states of aircraft, such as pitch and roll [21], descent angles [22] and lateral drift [23] for fixed-wing aircraft and to perform altitude control for automatic landing [13,14]. Furthermore, the study of the feasibility and concept of using optical flow with thermal imaging for navigation has been demonstrated in [24,25].

The paper is organised in 10 sections. Section 3 outlines our previous works and the motivation for this study. Section 4 presents the $I^{2}A$ in one and two dimensions. Section 5 and Section 6 outline our hardware and software architectures. Section 7 considers our assessment methodology, including the flying platform, two experimental sites and its conditions. Section 8 and Section 9 report and analyse the collected data from the flights. Section 10 outlines the lessons learnt and future research directions.

## 3. Contributions

Previous studies by us [26,27] have started to systematically explore the concept of airborne thermal flow. These works utilised the LK in OpenCV. Additionally, the characteristics of thermal flow over 24 h were investigated and compared to the output of the visible-light-based optical flow sensor. One of the lessons learned from [27] was that thermal flow from LK performed poorly from midnight until just before sunrise due to the much lower contrast in thermal data long after the sun goes down.

While the LK technique yields reliable results with relatively low computational demand, it suffers from sensitivity to noise due to the requirement to compute derivatives and the need to find distinct features between frames even when contrast is low. As a result, the implementation of thermal flow based on the LK suffered in a cold-soaked environment with much less contrast in thermal images. To solve this problem, considering robustness to noise and suitability for sub-pixel movements for airborne applications, dense techniques such as the $I^{2}A$ [28] might sometimes be a better alternative to the LK in lower-contrast frames such as in cold-soaked environments.

The $I^{2}A$ was first proposed by Srinivasan in [28], which demonstrated its robustness to noise, and the fact that it does not require feature detection and tracking between frames or high-order spatial or temporal derivatives of images. Our version of the $I^{2}A$ was tailored for airborne applications with the addition of predicted motion during the flight to increase precision. Additionally, we aimed to explore the use of the $I^{2}A$ with low-resolution and low-contrast thermal images.

Furthermore, our $I^{2}A$ implementation utilised full radiometric 14-bit frames from the thermal sensor, while the LK in OpenCV only accepts 8-bit scaled intensity images. This means that the $I^{2}A$ had an initial advantage of obtaining 6 more bits depth from its input, potentially outperforming the LK while not necessarily being the superior algorithm for the situation. On the other hand, the LK in OpenCV is very well known for its accuracy and has become a “go-to” implementation in real-time applications. As a result, it is still valid to evaluate the $I^{2}A$ against the OpenCV implementation of LK as well as the PX4Flow.

## 4. Optical Flow Computation

The $I^{2}A$ has demonstrated its effectiveness when computing optical flow from an image plan, which is much less computationally demanding [28], and the $I^{2}A$ is best with small movement changes in images [29].

The $I^{2}A$ is used to compute the motion of one image or subimage with respect to another [30]. It estimates the distance between f(x,y,t) and f(x,y,t+1), across an observation window of arbitrary size, shape and spatial weighting, relative to the deformation between f(x−k,y,t) and f(x+k,y,t). For small displacements of the sensor, it is assumed that the input f(x,y,t+1) is approximated by $\hat{f(x,y,t)}$, a weighted linear combination of f(x,y,t) and f(x±k,y,t)

(1) $$\hat{f(x,y,t+1)}=f\left(x,y,t\right)+\frac{v_{x}}{2k}\left(f(x+k,y,t)-f(x-k,y,t)\right)$$

where $v_{x}$ is the angular velocity in pixels per frame shift, *k* is a reference shift in pixels that is small, but larger than any expected motion, and $v_{x}/2k$ specifies the deformation of f(x,y,t) normalised with respect to the distance between f(x−k,y,t) and f(x+k,y,t), or 2k pixels. For the constraint of interpolation to apply, the value of $v_{x}/2k$ will range between −1.0 and 1.0. Within an observation window psi, $v_{x}$ is solved by minimising the mean squared error between $\hat{f(x,y,t+1)}$ and f(x,y,t):

(2) $$\psi \left(x,y\right)⊗\left[{\left[f\left(x,y,t\right)-\hat{f(x,y,t+1)}\right]}^{2}\right]=0$$

and taking a derivative of (2) with respect to $v_{x}/2k$ yields the expression

(3) $$\frac{v_{x}}{2k}=\frac{\left(\psi \left(x,y\right)⊗\left[\left(f(x,y,t+1)-f(x,y,t)\right]\right)\left(\psi ⊗\left(f(x+k,y,t)-f(x-k,y,t)\right)\right)\right)}{\psi \left(x,y\right)⊗\left[{\left(f(x+k,y,t)-f(x-k,y,t)\right)}^{2}\right]}$$

where $v_{x}/2k$ is the normalised position of f(x,y,t+1) between f(x−k,y,t) and f(x+k,y,t), and thus $v_{x}$ is the shift between f(x,y,t+1) and f(x,y,t) in pixels.

To reduce aliasing caused by high-frequency components, the images are passed through a low-pass filter, which can be a Gaussian or a square convolution kernel, before computing optical flow.

### Extension to Two Dimensions

The theory can be extended to compute optical flow in two dimensions. Assuming that the motion has two degrees of freedom, with a small shift, the shifted image can be presented as:

$$\begin{array}{cc} \hat{f(x,y,t+1)} & =f\left(x,y,t\right)+\frac{v_{x}}{2k}\left(f(x+k,y,t)-f(x-k,y,t)\right)\\ & \frac{v_{y}}{2j}\left(f(x,y+j,t)-f(x,y-j,t)\right) \end{array}$$

Similar to Equation (3), the parameters $v_{x}$ and $v_{y}$ can be solved by setting $\frac{\partial v_{x}}{\partial v_{y}}$ and $\frac{\partial \;v_{y}}{\partial v_{x}}$ to zero.

The resulting simultaneous equations may be expressed in matrix form, as follows:

(4) $$\left[\begin{array}{cc} A^{2} & AB\\ AB & B^{2} \end{array}\right]\left[\begin{array}{c} v_{x}/2k\\ v_{y}/2j \end{array}\right]=2\left[\begin{array}{c} AC\\ BC \end{array}\right]$$

where

$$\begin{array}{ccc} A & = & \psi \left(x,y\right)⊗\left(f(x+k,y,t)-f(x-k,y,t)\right)\\ B & = & \psi \left(x,y\right)⊗\left(f(x,y+j,t)-f(x,y-j,t)\right)\\ C & = & \psi \left(x,y\right)⊗\left(f(x,y,t+1)-f(x,y,t)\right) \end{array}$$

Matrix inversion is required at each point in the images generated by the expressions defining the matrix coefficients, and after each image has been convolved with a two-dimensional kernel, ψ, which acts to localise each motion computation. Gaussian ψ kernels were used throughout the flight tests.

## 5. Hardware Implementation

**Figure 1:**
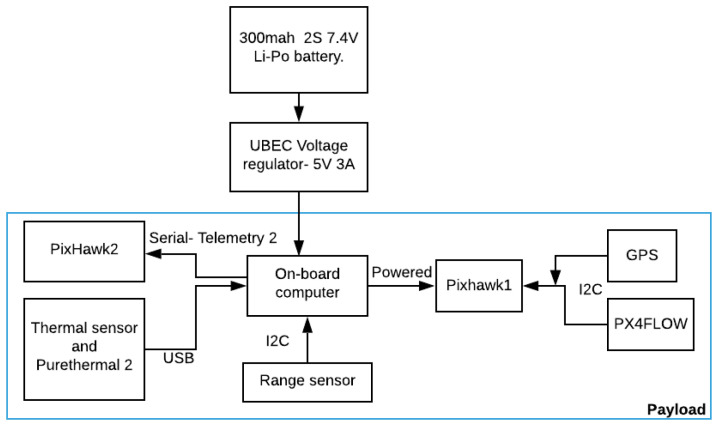
Hardware implementation block diagrams of the system.

This section describes the hardware system used in the experiment. All the components had to satisfy three constraints: low in cost, light in weight and small in size. Figure 1 shows a block diagram of all the components of our system. A lithium-polymer battery was used to power the system via a 5 V power supply voltage regulator to maintain constant voltage and current throughout the experiment.

**Figure 2:**
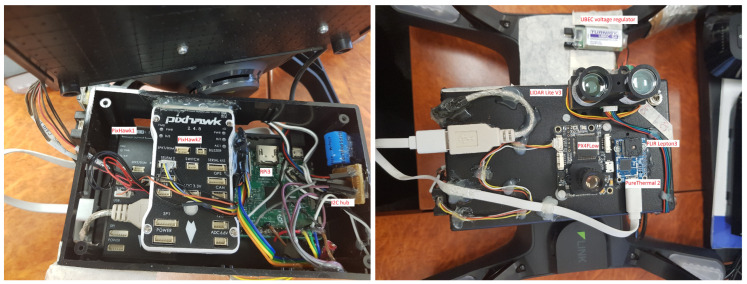
Inside (on the **left**) and outside (on the **right**) of the system.

Figure 2 shows our constructed payload in the housing frame, with components labeled in red.

### 5.1. Thermal Sensor

The FLIR Lepton 3 (Teledyne FLIR LLC., Wilsonville, OR, USA) was chosen in this study due to its light weight and low cost. The FLIR Lepton 3 is an uncooled LWIR thermal sensor with a 56° field of view [31]. The sensor has a low angular error of 0.03°, which is adequate, without any calibration needed [26]. Additionally, the sensor also satisfies both weight and size constraints at 0.9 g and 11.8 mm × 12.7 mm × 7.2 mm in size. The sensor can output 14-bit 160 × 120 radiometric resolution thermal images at 8.7 HZ.

#### Flat Field Correction

The thermal sensor comes with a built-in shutter with Flat Field Correction (FFC) for stationary usage. The FFC compensates for errors that build up over time during operation. The FFC is essential when the sensor captures the same scene for a prolonged period to prevent ghosting [31]. During the FFC process, the sensor freezes for a small amount of time depending on the model (0.3–2 s), which is undesirable for navigation applications. Since the Lepton is mounted on a constantly moving aircraft, it is essential to disable the FFC to achieve continuous inputs.

### 5.2. Interfacing with the Lepton Sensor

The Lepton is integrated on the Purethermal 2 board. The board weighs 50 g, with the dimensions of 30 mm × 18 mm. The board uses its own integrated circuit with an integrated ARM microprocessor, which is capable of executing the Lepton commands by itself, thus freeing up some of the processing that would otherwise be done by the main computing system, the Raspberry Pi 3. The Purethermal 2 board interfaces with the Raspberry Pi via a USB connector.

#### Range Sensor

A LIDAR lite v3 (Garmin Ltd., Lenexa, KS, USA) [32] was used in this study due to its light weight of only 22 g and low power consumption. The purpose of the range sensor is for post-flight altitude verification.

### 5.3. Onboard Processor

The Raspberry Pi 3 (Pi 3) (Raspberry Pi Foundation, Cambridge, UK) was used in this study to obtain and save 14-bit raw thermal images from the Lepton 3 for later processing. The Pi 3 satisfies both weight and size constraints for small aircraft applications.

### 5.4. PixHawk and PX4Flow

The PixHawk was powered by the Pi 3 via “Telemetry 2” connection. The PX4Flow interfaces with the PixHawk via the I2C communication protocol. Data “Ulog” files were saved on the PixHawk, which captured the optical flow signals from the PX4Flow.

## 6. Software Implementation

**Figure 3:**
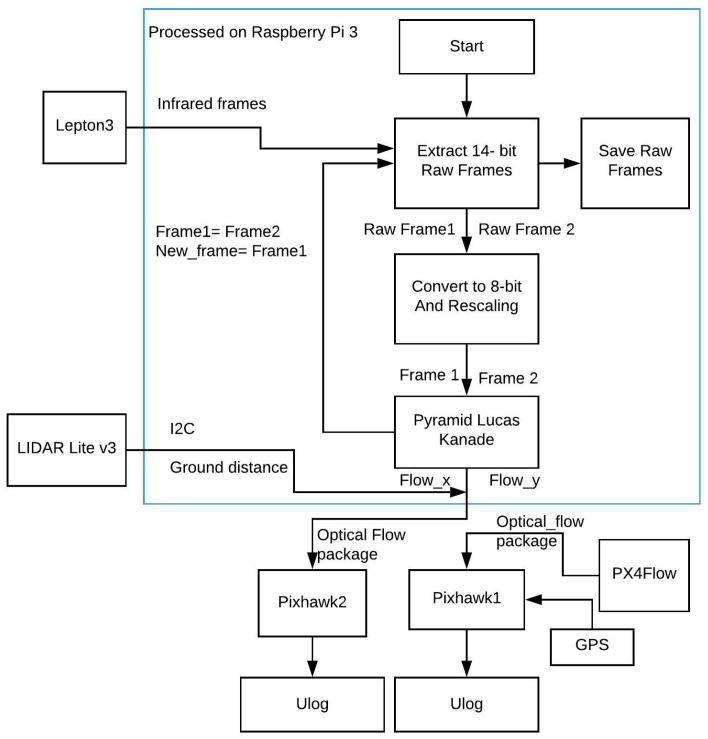
Software algorithm block diagram.

Figure 3 shows the structure of our payload. The Pi 3 is the main computer of the system, requesting raw 14-bit data from the Lepton 3 in the beginning. The raw 14-bit data were saved for later processing. Each consecutive 14-bit frame then will be down-scaled to 8 bits while maintaining a common scale with the technique in [27]. The two processed frames were processed with the LK in OpenCV to determine the 2D optical flow vector (flow_x, flow_y). The ground distance was also received from LIDAR, and it is sent along with the optical flow vector to the PixHawk2 via the “OPTICALFLOWRAD” MAVLINK package to be saved in Ulog format. The PX4Flow data were also saved on the PixHawk1.

In this study, the system was constructed with OpenCV version 4.5.5, Numpy version 1.19.2 and Python version 3.8.

### 6.1. Lucas–Kanade Algorithm in OpenCV

The LK implementation in OpenCV uses Shi–Tomasi [33] corner feature detection to identify distinguishable features across two images. The LK optical flow technique operates based on three assumptions [15]:

- Brightness constancy: The contrast should not differ between two frames.
- Small movements: The displacement between two frames should not be too large.
- Spatial coherence: The neighbouring pixels should move together and have the same motion across two frames.

The three conditions must be met in order to compute the optical flow field between two frames with the LK and the $I^{2}A$.

### 6.2. Automatic Gain Control

In modern thermal sensors such as the FLIR Lepton 3, the Automatic Gain Control (AGC) is turned on by default to give to the user the most detail when the average temperature of the scene is changing. When the sensor first captures radiometric thermal data, the data are in a 14-bit depth format, which are “raw” data. However, the raw 14-bit data must be converted down to 8 bits to visualise them on electronic displays. Additionally, the LK implementation in OpenCV only accepts 8-bit input data for optical flow estimation. Hence, it is necessary to convert them from 14 bits to 8 bits.

**Figure 4:**
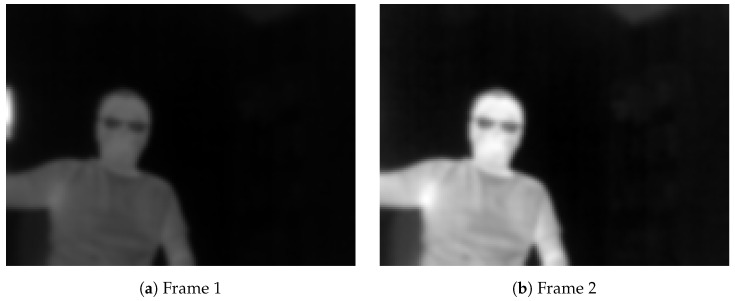
Automatic Gain Control (AGC) changes the contrast in an image when a hot cup moves into a scene: (1)–(2).

By default, the AGC built into the sensor is responsible for this. However, a problem arises when there is a drastic change in the scene temperature, when a significantly hotter or cooler object enters or exits the scene. One example, in Figure 4, shows two 8-bit frames taken continuously when a hot cup is moving out of the scene when processed with AGC.

Additionally, Table 1 shows the average, 10% and 90% percentile for the pixel intensity of the images shown in Figure 4. It is clear that the pixel intensity of an image changes dramatically when a very hot or cold object enters or exits the scene due to the AGC.

The AGC was designed to show the maximum possible dynamic range of the image, which is good for inspection purposes. However, this may cause problems for many feature matching algorithms due to the drastic change in contrast between images. Additionally, rescaling 14-bit images with the AGC also violates the first condition of the LK technique: brightness constancy.

#### 6.2.1. Rescaling Technique

This section summarises our technique from [27] to convert 14-bit raw thermal images to 8-bit images while maintaining the contrast between two images.

**Figure 5:**
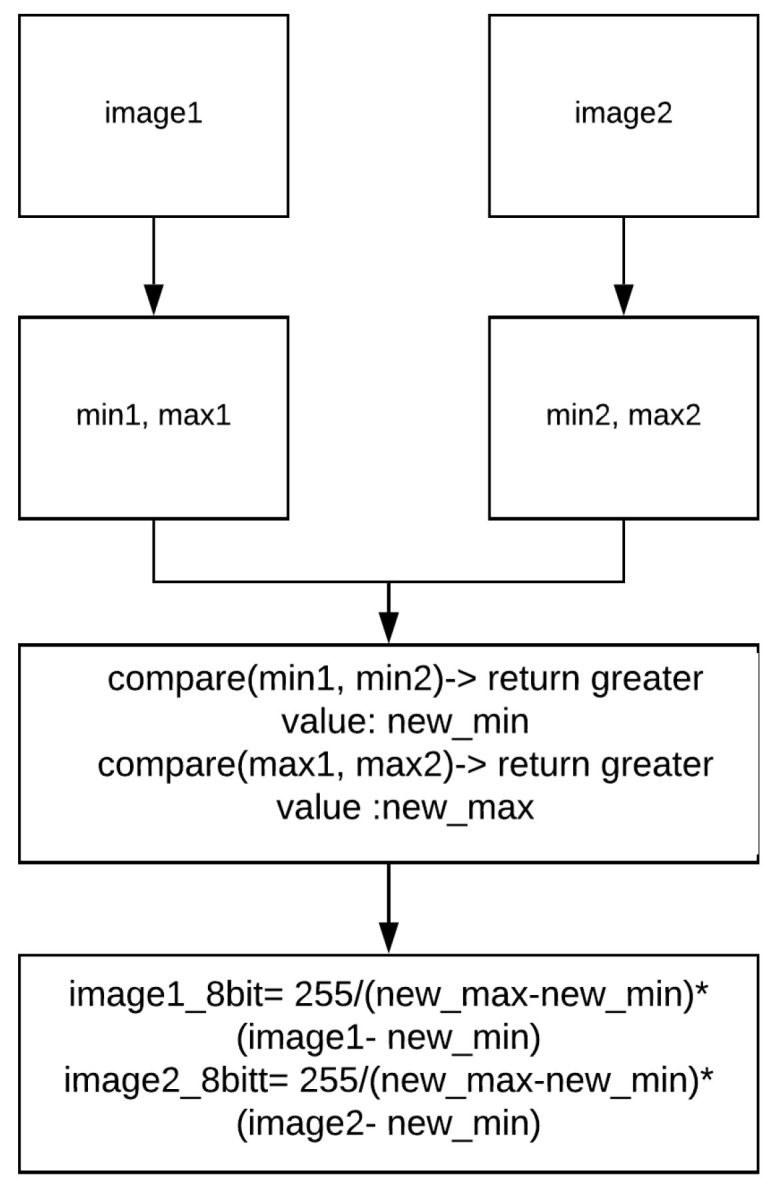
A pair of images with the same scaling value.

Our technique takes two 14-bit images as input, and then rescales these two frames based on their maximum and minimum pixel intensity. Figure 5 shows the conversion of a pair of 14-bit images, image1 and image2, to two 8-bit images.

**Figure 6:**
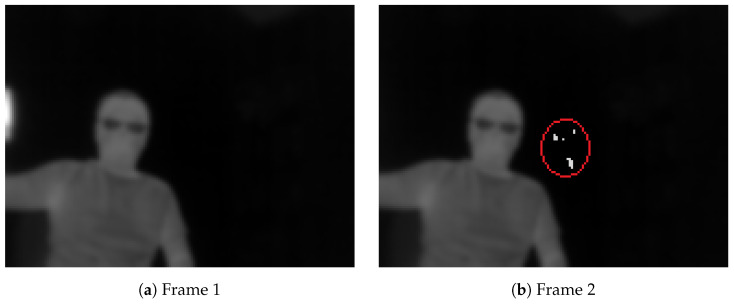
A pair from Figure 4 with our technique. In this extreme case, there are small artefacts, which are circled in red.

Figure 6 shows the rescaled images with our technique from Figure 4. The result shows that the brightness does not change but there are small artefacts in the second image in this case.

**Figure 7:**
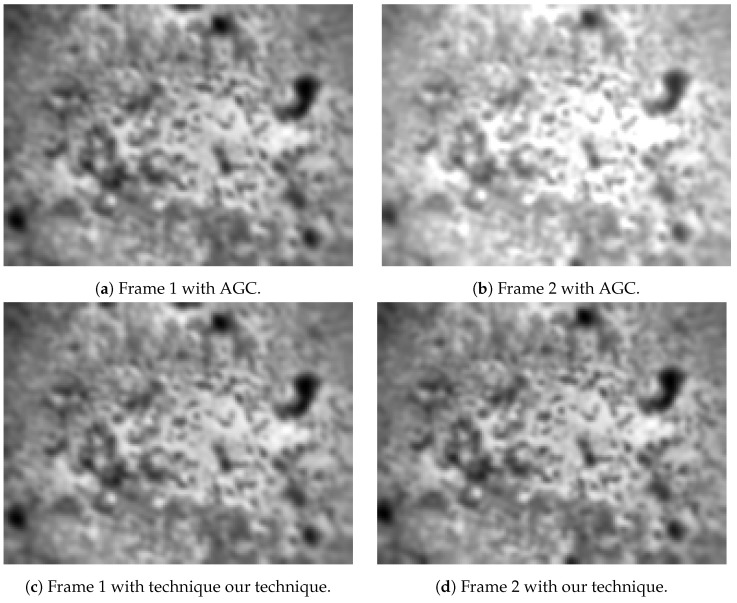
Two captured consecutive frames with AGC enabled (**a**,**b**) and with technique (**c**,**d**).

Figure 7 shows a pair of images captured from the flight, processed with AGC and our technique. Visually, a pair with AGC cannot be used for optical flow estimation due to corresponding pixels having different gains applied to their read-out. On the other hand, when applying our technique, the second rescaled image is able to maintain its contrast. Additionally, the undesired artefact effect is too small to be visually detected.

Table 2 shows the average, 10% and 90% percentile pixel intensity from images shown in Figure 7. It is clear that the same brightness is maintained across two images for optical flow estimation.

Good, distinct features were found based on the Shi–Tomasi algorithm [33]. The output of the algorithm is single displacement vectors in two dimensions as a median of all good points found. Table 3 shows the parameter settings for LK and Shi–Tomasi algorithms in OpenCV.

#### 6.2.2. Benefit of 14-Bit Implementation with the I^2^A

Table 4 shows the pixel intensity of a pair of 14-bit images, showing that the brightness is consistent across both images. Hence, there is no need to apply the conversion techniques in Section 6.1 while using full radiometric 14-bit data.

Besides bypassing the troubled AGC and rescale techniques, utilising full 14-bit radiometric data directly can provide better results compared to their 8-bit version counterpart [9]. Additionally, the team in [6] also showed that using full 14-bit radiometric data makes the algorithm more resilient to the occasional absence of data due to the availability of an additional 6 bits of data that would otherwise be lost during the conversion technique.

In this experiment, the shift values are 4 pixels in both X and Y displacements and the chosen kernel is a 9 × 9 Gaussian.

## 7. Assessment Methodology

This section shows how the tests were conducted, including the site of the experiment, flight plans, flying platform, weather conditions at the site and how the signals were analysed.

### 7.1. Flying Platform

The payload was mounted underneath the 3DR SOLO (3DR, Berkeley, CA, USA). The now obsolete SOLO was available and had the capacity to carry a payload of up to 500 g with a tolerable flight time of 10 min.

### 7.2. Field Experiment

**Figure 8:**
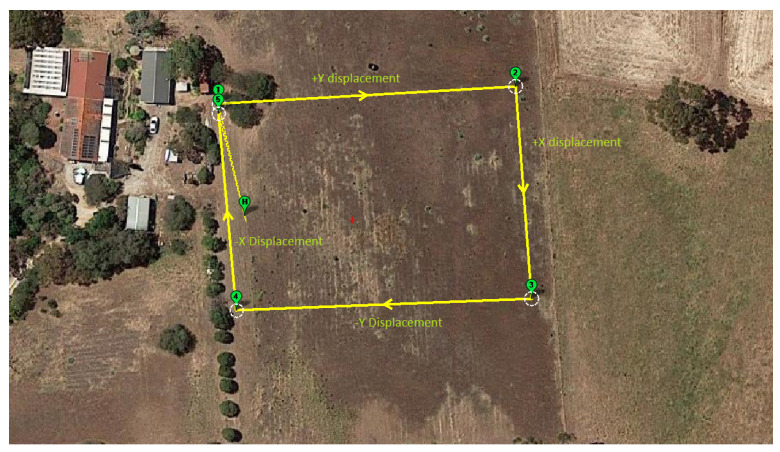
Flight plan in mission planner.

The SOLO was programmed to fly one square lap at a constant height of 8 m with target velocity at 8 m/s. Figure 8 shows the flight path in this experiment. The SOLO took off at point H, flew to point (1)-(2)-(3)-(4)-(5) and then landed at (5).

We carried out two flight trials in this study. The first trial was during a normal sunny day in autumn, while our second trial was during a cold and foggy day several months later in winter. Both trials were performed at the same field with the same flight plan.

The purpose of the first trial was to compare the performance of the LK, the $I^{2}A$ and the PX4Flow during normal conditions: on a sunny, clear-sky day with high thermal contrast. The second trial aimed to compare them in a cold-soaked, lower-contrast environment.

Table 5 shows the field conditions, including min and max temperatures, temperature and weather conditions, at the time for each experiment [34].

It was expected that the PX4Flow and the LK would perform worse in cold-soaked conditions, as learned from our previous study in [27]. Additionally, we also used the same flight plan at the same experimental site to evaluate LK and $I^{2}A$ performance in the two experiments.

#### 7.2.1. Experiment 1: High Thermal Contrast Condition

**Figure 9:**
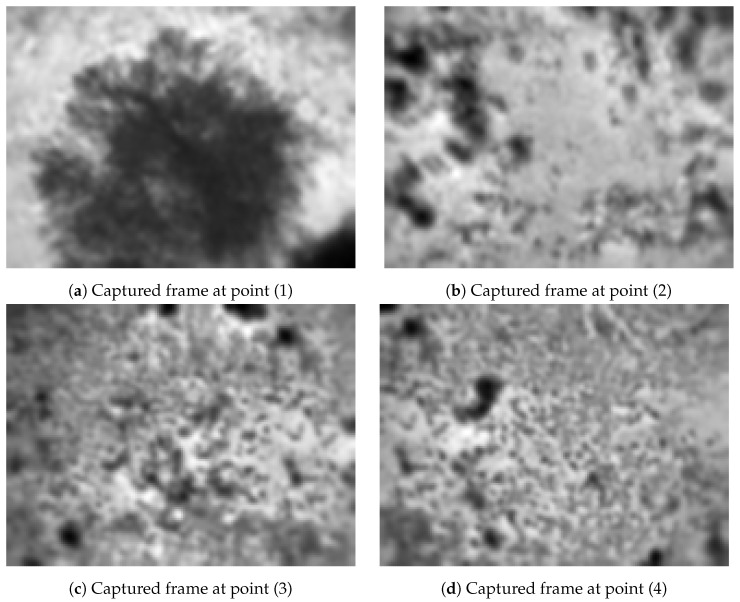
Processed 8-bit thermal frames of some interesting points in Experiment 1.

The first test was conducted at 11am, during a clear and sunny autumn day at the site. Figure 9 shows some thermal images of the site taken from the SOLO; all the images are in 8 bit and were converted from 14 bits with the same scale as described in Figure 5.

#### 7.2.2. Experiment 2: Lower Contrast Condition

**Figure 10:**
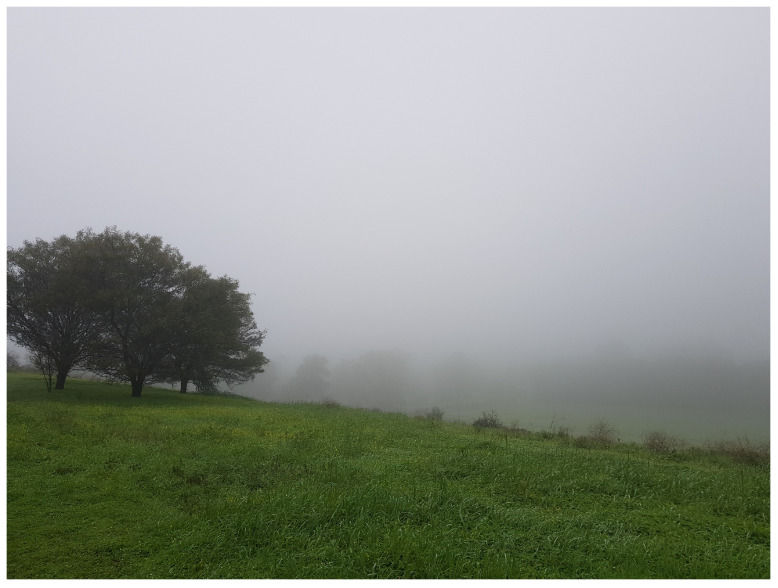
Field condition in Experiment 2.

The second trial was done in winter at 0900 h at the same location, using the same flight plan. Figure 10 shows the lack of sunlight and rainy and foggy condition of the site.

**Figure 11:**
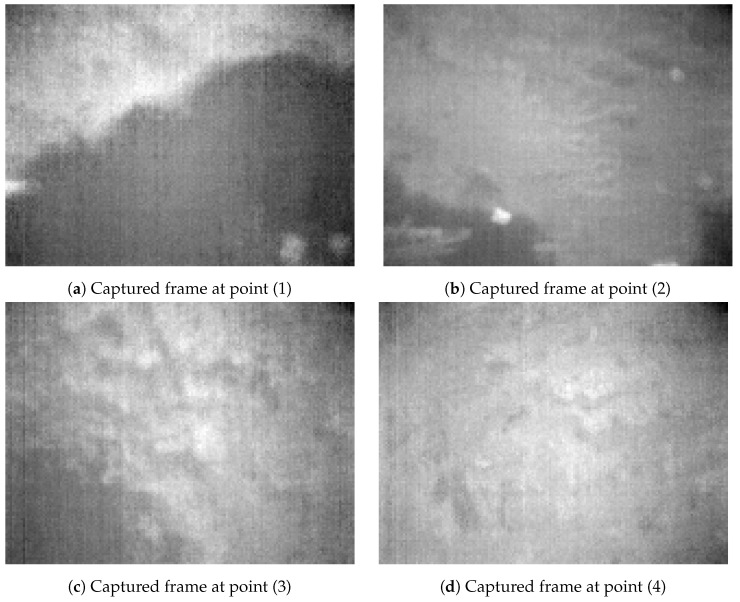
Processed 8-bit thermal frames at same locations as in Figure 9, in Experiment 2.

Figure 11 shows captured and processed 8-bit thermal frames at four interesting points during Experiment 2, at approximately the same location as in Experiment 1. It indicates that the thermal frames in Experiment 2 contained much less contrast, details and dynamic ranges compared to Experiment 1.

### 7.3. Signal Analysis

To evaluate the performance of the LK, the $I^{2}A$ and the PX4Flow sensor, the output signals of each technique were compared to each other in both X and Y displacements. Cross-correlation processing was applied to determine how closely the two signals matched each other. High and positive cross-correlation indicates that the two signals are well matched.

## 8. Results

This section shows the optical flow measurements from the two flight tests in both X and Y axes. We also used our collected data from our previous paper [27].

### 8.1. Experiment 1

This section shows the resulting signal for our flight test in X and Y displacements for the $I^{2}A$, the LK and from the PX4Flow.

**Figure 12:**
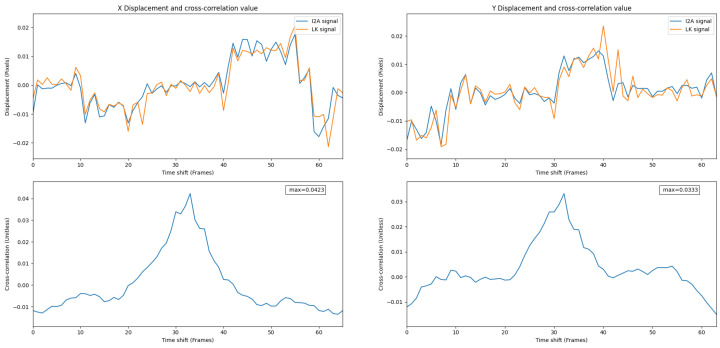
Overlay of the $I^{2}A$ and the LK signals in Experiment 1. A high positive correlation value shows a strong relationship between these two signals.

Figure 12 shows the overlaid signals and the cross-correlation value of the LK and the $I^{2}A$. Given the high value of cross-correlation, the $I^{2}A$ performed as well as the LK during the test in both the X and Y displacements.

**Figure 13:**
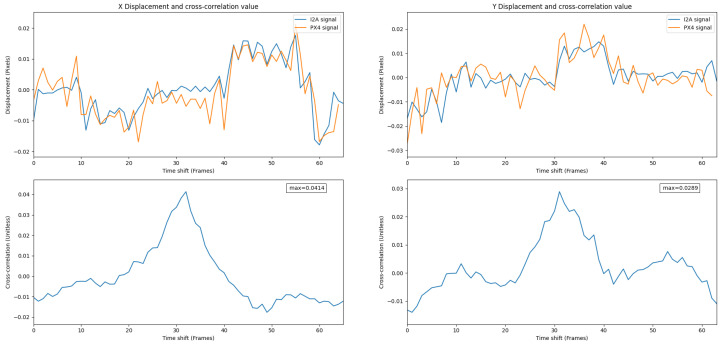
Overlay of the PX4Flow and the $I^{2}A$ signals in Experiment 1. A high correlation value shows a strong relationship between these two signals.

Figure 13 shows overlaid signals and their cross-correlation values for the PX4Flow and the $I^{2}A$. A high correlation value shows a strong relationship between these two signals.

**Figure 14:**
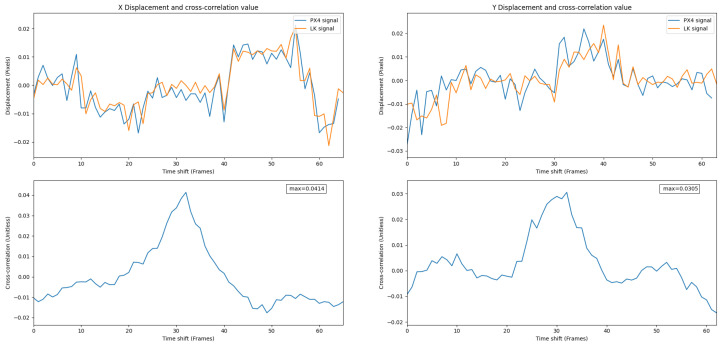
Overlay of the PX4Flow and the LK signals in Experiment 1. A high correlation value shows a strong relationship between these two signals.

Figure 14 shows overlaid signals and their cross-correlation values for the PX4Flow and the LK. A high correlation value shows a strong relationship between these two signals.

**Figure 15:**
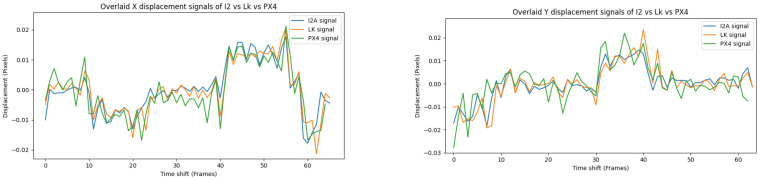
Overlay of the $I^{2}A$, the LK and the PX4Flow signals in Experiment 1.

Figure 15 shows the three signals—the $I^{2}A$, the LK and the PX4Flow—over X and Y displacements.

### 8.2. Experiment 2

In Experiment 2, the PX4Flow did not work, while thermal flow with the LK and the $I^{2}A$ was functional.

**Figure 16:**
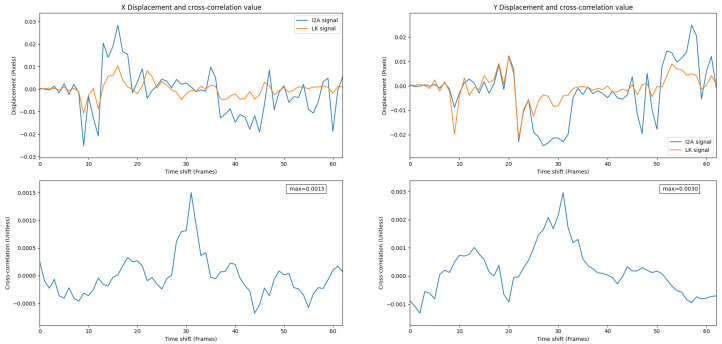
$I^{2}A$ and the LK signals in cold-soaked conditions, Experiment 2. A weaker correlation value shows a weaker relationship between signals compared to Experiment 1.

Figure 16 shows thermal flow measurements from the LK and the $I^{2}A$. The results clearly indicate that the $I^{2}A$ works better than the LK in this trial. While the LK can keep up with the $I^{2}A$ at some points during the flight, the $I^{2}A$ still yields some flow measurements while the LK yields nothing. At point (1) and (2) in Figure 11, the $I^{2}A$ and the LK produce comparable results since the frame contrast is still high. However, while the contrast is low at point (3) and (4), the LK cannot keep up with the $I^{2}A$. Hence, the $I^{2}A$ has the advantage in cold-soaked, low-contrast conditions.

### 8.3. Aperture Problem

The aperture problem refers to the phenomenon that causes one-dimensional spatial structures such as a bar, line or edge to be determined ambiguously when viewing from a small hole, when the motion is not known [35].

**Figure 17:**
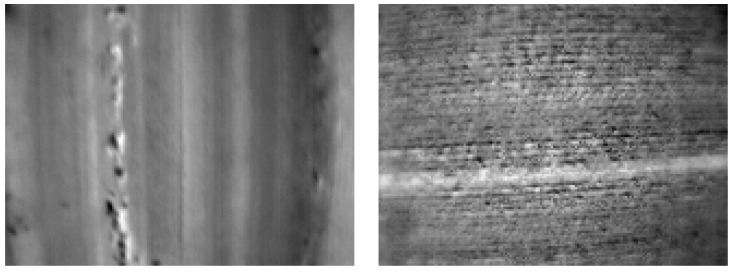
Thermal images of the main road (**left**) and the wheat field (**right**) at 1400 h.

Two parts of our dataset at 1400 h from our previous work in [27] were used to test the performance of the $I^{2}A$ in a scene when the aperture problem was prominent. We used sequences of the main road and wheat field with a strong vertical and horizontal line, as shown in Figure 17.

**Figure 18:**
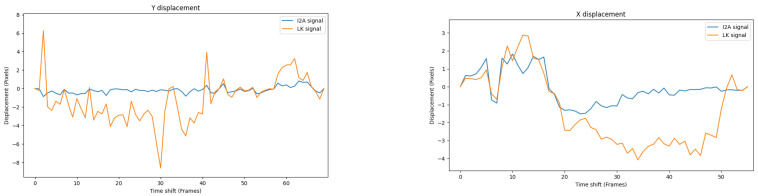
The $I^{2}A$ and the LK signals in Y and X displacements above the main road (**left**) and the wheat field (**right**). It is clear that the $I^{2}A$ does not work in these scenarios.

Figure 18 shows a comparison of Y displacement over the main road and the X displacement over the field of the $I^{2}A$ and the LK.

From Figure 18, the $I^{2}A$ suffers from the aperture problem while the LK does not. This issue potentially limits the use of the $I^{2}A$ in some scenarios.

## 9. Discussion

The greatest weakness of simple optical flow algorithms based on spatio-temporal gradients is their inability to deal with the aperture problem or to detect information deficiencies in the image. This problem is entirely based on how they were originally formulated, and is not particularly fundamental, although it does deviate from the ideal of massive Single Instruction Multiple Data (SIMD) processing of the entire image in a single pass. In this sense, the aperture problem seems to be a reasonably manageable problem, since there is no particular reason that a salience operator could not be run across the entire image also using SIMD instructions, and then used as a gate to determine which data are likely to be valid. This is well travelled ground in the literature from decades ago [36,37].

Outdoor environments are difficult to control. However, it seems likely that under cold-soaked conditions, such as very late at night, where noise is more apparent in thermal images [27], the $I^{2}A$ might have some advantages, as shown in Figure 16.

In general, agreement between the $I^{2}A$ and LK and PX4Flow shows that both the dense and sparse optical flow techniques can be used reliably with low-resolution thermal data for airborne applications.

## 10. Conclusions

The results have shown that the $I^{2}A$ is capable of computing optical flow reliably from low-resolution thermal imagery, compared to the LK technique and the PX4Flow. Additionally, the $I^{2}A$ performs better than the LK in lower contrast and higher noise during colder conditions.

The $I^{2}A$ can take advantage of new-generation compact systems with more powerful graphics processing units, such as the Nvidia Jetson, that are capable of SIMD processing. Moreover, the $I^{2}A$ is robust to noise, while the “aperture problem” is a considerable issue. Hence, we expect the $I^{2}A$ to continue to work well compared to the LK during the day and better during the night due to the characteristics with noise and sensitivity. It is the case, however, that the LK has a built-in test of the quality of the result, based on the number of features.

The drone was programmed to fly at constant height and speed in this study; a fixed “shifting value” worked well in this scenario, but might not work well in situations where the altitude or velocity changes over time. Therefore, the shifting value should be able to update itself to respond to the changes in height and velocity of the UAVs.

Further study will focus on variations of these algorithms that are better tuned to the aerial environment for fixed wing flight and to deal with the aperture problem. Additionally, further study will also investigate the deep learning approach for thermal flow.

## Figures and Tables

**Table 1:** Pixel intensity values with Automatic Gain Control (AGC) of two images shown in Figure 4.

	Pixel Intensity	Mean	90% Percentile	10% Percentile
AGC	Frame 1	71	174	20
	Frame 2	55	132	17

**Table 2:** Pixel intensity values of a pair of 8-bit images with AGC (first two rows) and our technique (last two rows).

	Mean	90% Percentile	10% Percentile
Frame1_AGC	139	185	95
Frame2_AGC	188	238	144
Frame1_Same	139	185	94
Frame2_Same	139	184	94

**Table 3:** Setting parameters for LK optical flow and Shi–Tomasi corner detection algorithm.

Feature Detection Settings	Maximum corners	1000
	Quality level	0.02
	Minimum distance	5
	Block size	5
LK Settings	Window size	(15,15)
	Maximum pyramid level	2
	Search termination count	10
	Search termination ϵ	0.03

**Table 4:** Pixel intensity values of two unprocessed 14-bit images, which shows that they both have approximately the same contrast and overall pixel brightness.

		Mean	90% Percentile	10% Percentile
14-bit	Frame 1	30,070	30,307	29,759
	Frame 2	30,100	30,484	29,773

**Table 5:** Weather conditions at the experimental sites.

	Experiment 1	Experiment 2
Min temperature	17 °C	5 °C
Max temperature	31 °C	11 °C
Temperature at the time of flying	27 °C	9 °C
Field condition at the time of flying	Clear, sunny	Foggy with light rain

## Data Availability

Not applicable.

## Abbreviations

The following abbreviations are used in this manuscript:

LWIR	Long-Wavelength Infrared
AGC	Automatic Gain Control
FFC	Flat Field Correction
*I^2^A*	Image Interpolation Algorithm
UAV	Unmanned Aerial Vehicle
LK	Lucas–Kanade Algorithm
